# A Concise Review on Qualitative Research in Dentistry

**DOI:** 10.3390/ijerph18030942

**Published:** 2021-01-22

**Authors:** Hollis Haotian Chai, Sherry Shiqian Gao, Kitty Jieyi Chen, Duangporn Duangthip, Edward Chin Man Lo, Chun Hung Chu

**Affiliations:** Faculty of Dentistry, The University of Hong Kong, Hong Kong 999077, China; htchai89@hku.hk (H.H.C.); sherryg@hku.hk (S.S.G.); chenjy679@mail.sysu.edu.cn (K.J.C.); dduang@hku.hk (D.D.); hrdplcm@hku.hk (E.C.M.L.)

**Keywords:** qualitative research, quantitative research, dentistry

## Abstract

Qualitative research collects non-numerical data that explores human behaviour, attitudes, beliefs and personality characteristics unamendable to quantitative research. The qualitative research questions are open-ended, evolving and non-directional. The study design is flexible and iterative. Purposive sampling is commonly used. The sample size is determined by theoretical saturation. Data collection is generally through in-depth interviews, focus groups and observations. Qualitative research commonly uses thematic analysis and framework analysis, although there is no consensus on analysing qualitative data. The reporting format can be comprehensive, a summary, developmental or selective, subject to the research question. Qualitative research’s potential functions are to describe the form or nature of what exists (contextual), to examine the reasons for or associations between what exists (explanatory), to appraise the effectiveness of what exists (evaluative), and to aid the development of strategies (generative). Qualitative research can be time consuming to conduct because it explores evolving questions; difficult to generalise because it recruits limited participants; and arduous when it comes to making systematic comparisons because responses are subjective. However, qualitative research can provide depth and detail, create openness, simulate people’s individual experiences and avoid pre-judgements. This concise review provides an overview and suggestions for dental researchers when conducting a qualitative study.

## 1. Introduction

The vast majority of dental research has been conducted in quantitative methodologies in nature [1]. Over the last few decades, along with the rise of qualitative studies conducted in health services research, dental researchers recognized the significant role of qualitative research in dentistry. Qualitative research provides a new perspective of perceiving and exploring questions relevant to dental knowledge and clinical practice rather than quantitative methods alone [2]. It refers to a set of methodological approaches to interpret and understand social phenomena by exploring the subjects’ experience, behaviours, perspectives and characteristics within their natural setting. The form of realization can be interviews, fieldnotes, conversations, recordings, photographs and memos [3]. Thus, the data obtained in qualitative research are usually via text, audio or visual [4]. Although qualitative research has experienced a rise in the dental area, mostly in dental public health [5], both the awareness and understanding of qualitative research are still relatively limited among dental researchers. To help dental professionals who desire qualitative research and want to conduct a constructive and methodologically rigorous qualitative study, this paper provides an overview and suggestions for dental researchers in conducting a qualitative study.

## 2. Quantitative and Qualitative Research

Unlike quantitative research that aims to establish generalizable facts under controlled experimental settings, qualitative research primarily aims to provide a deeper, contextualized understanding of social phenomena through intensive studies of particular cases under natural settings [6]. The qualitative research domains are compared with quantitative research in Table 1. Nevertheless, qualitative research should not be regarded as the opposite of quantitative research. Because of the differences in research nature, they address different research aspects based on the research problem [1].

## 3. Conducting a Qualitative Research

**Figure 1:**
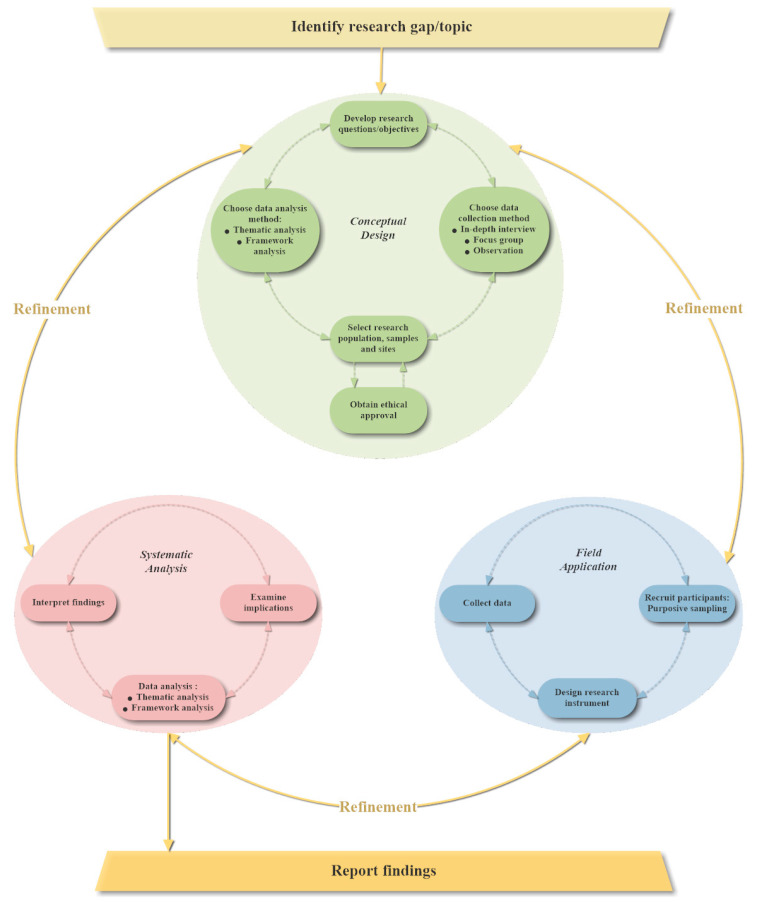
Research cycle of qualitative studies (Adapted from Ritchie et al., 2003; Hennink et al., 2020 [3,8]).

Qualitative research typically has been used to explore unanticipated issues and involves an element of unknown [7]. Qualitative studies’ central tenants are interpreting the meaning people attached to their experience and studying people in their natural settings. Different researchers have different beliefs of the social reality and philosophical traditions behind qualitative research. Moreover, researchers themselves’ position within the study context and their relationship with study participants can be varied along with the study’s purposes changing over the course of the research. These fundamentals of qualitative research have decided there is no single standard way of conducting it [3]. Figure 1 is a research cycle for conducting qualitative studies. Iterative designing and continuous reviewing are essential for effective qualitative research. Detailed information for each step will be explained in further context.

The starting point is usually the researchers’ intention to explore a social phenomenon, which will then be broken down into specific research questions or objectives. The conceptual design period involves tasks such as choosing the data collection method; selecting research populations, samples and sites; and choosing the data analysis method. After obtaining ethical approval, the study logically moves from conceptual design period to its field application period. Field application comprises core elements including participant recruitment, research instrument design and data collection. Through systematically analysing the collected data and making explanatory interpretations, the findings can be generalised [8]. The relationships among these three periods are iterative, each should inform and be informed by the other two. Qualitative research provides the flexibility to compare the inductive findings with the original conceptual design to discern the contribution and make refinements. Therefore, researchers are recommended to leave enough time for reflection so they can address emerging issues throughout the qualitative research process [3].

Since there is a lack of a gold standard in conducting qualitative research, it is vital to ensure the quality and rigor of the research. Several Appraisal frameworks have been generated to assess the quality of qualitative research [9]. One of the frameworks in appraising qualitative research in healthcare systems is the “Quality in Qualitative Evaluation: A framework for assessing research evidence”, produced by Spencer and collogues on behalf of the UK cabinet office [10]. This framework contains four guiding principles and 18 appraisal questions to assist quality assessment. These frameworks should be applied flexibly and avoid being over-prescriptive to achieve an informed judgement on qualitative research [5].

### 3.1. Identify the Research Topic and Define the Research Questions

A clearly identified research topic is essential for a qualitative study. There are several pathways to identifying a research topic. Usually, the starting point is the researcher’s interests or hunches from under-explored areas within their professional field. Researchers can also take inspiration from their daily life activities or the population who has a direct experience on the issue, which is the so-called “user involvement” [11]. Another way of identifying a research topic is called commissioned research, which refers to those studies of which the commissioners (i.e., stakeholders, funder) identifies the initial ideas.

The initial research topic will be narrowed down to more specific and detailed research questions/objectives through reviewing existing theory and literature. For qualitative research, the research questions should be open-ended, evolving and non-directional, which often includes one central question and several sub-questions. Each defined central question can have a series of “sub-questions” following them. The sub-questions fall under the umbrella of the central research questions, but indicate any clarifications and parameters of the research. Defining clear and relevant research questions are essential for qualitative research since the data collection method and the steps that follow it all depend upon the research topic and questions [12]. Qualitative research also provides the facility of refining research questions during the data collection and analysis process.

### 3.2. Sampling Method

By contrast with statistical probability sampling in quantitative research, purposive sampling is the most robust sampling method in qualitative research. Units are chosen on purpose to reflect their particular characteristics relevant to the study topic [13]. Meanwhile, qualitative sampling is intended to cover all of the subject matter’s relevant key parameters and possess enough diversity within each criterion to allow in-depth exploration. The sample size in qualitative research is relatively small, which is determined by theoretical saturation [14]. This means the sampling will stop at the point when increasing the sample size would no longer contribute to new evidence. This scale of sample size facilitates the detailed exploration of each sample and maximizes the usefulness of the data collected [9].

The first step of purposive sampling is to decide the study population and the sample frame. The study population in qualitative research usually involves people at some stages, but it is also possible to include records, images and documents [15]. The next step is to set purposive selection criteria and prioritize them based on the research topic and questions. Since the context is already known, it is also necessary to decide the study’s locations to make it more salient to participants. Finally, it is helpful to design a sample matrix with the purpose of setting quotas for final participant selection. The process of drawing a sample matrix is basically mapping out previously determined selection criteria (vertically and horizontally) and assigning appropriate numbers of units in each yielded cell [3].

### 3.3. Data Collection

In-depth interviews, focus groups and observations are the most commonly used methods for data collection in qualitative research. The choice of different methods depends on a number of issues, such as the research topic, study population, the nature of data and practical issues such as accessibility, social context and the sensitivity of the subject matter [3]. A comparison of three data collection methods is displayed in Table 2. The different steps in conducting three data collection methods are displayed in Table 3.

#### 3.3.1. In-Depth Interview

The in-depth interview, one of the core qualitative research methods, is the most frequently used and well-established qualitative data collection method in healthcare settings [1]. Different from the well-structured interviews in quantitative research, in-depth interviews are usually semi-structured with several pre-planned open-ended questions and follow progress-based probing questions [16]. The power of an in-depth interview is allowing the research topic to be explored from a participants’ perspectives in depth and detail [17].

There is no single standard way of conducting in-depth interview, but it usually contains six steps [3]. Before an interview starts, it is best to establish an initial rapport with the interviewee. Informed consent should be obtained after introducing the scope of the research and ethical principles. It is also helpful to emphasize for the interviewee that there is no standard correct answer for each question. Therefore, they can be more relaxing and honest to express. Collecting contextual background information at the beginning of the interview is necessary for reference and to set the tone. The sequencing of asking questions during the interview should be from easy to difficult, from mapping to probing. Leading questions should be avoided [18]. The interviewer should stay in an empathic but neutral stance when pursuing the breadth and depth of the coverage topic. At the end of the interview, please thank the participants and check whether they want to add something that was not covered in the interview. Audiotapes and audio-video tapes are the most common ways to record data. Sometimes it is also valuable to take “fieldnotes” about observations and ideas during the interview to help with the later data analysis [19].

#### 3.3.2. Focus Group

The focus group is also a mainstream qualitative research method in the form of a group discussion on a particular research topic. This group discussion can be naturally occurring or composed by recruited participants [4]. A facilitator (usually the researcher) will be included in the discussion to moderate and monitor the process [20]. A focus group is suitable to use when capturing information generated through group interaction or when displaying a social context is needed. A focus group encourages participants to interact with each other within the collective context. This interactive dynamic process can trigger new ideas from the participants and encourage a deeper discussion of the research topic. The discussion group works synergistically to extract information from a series of issues in a relatively short time [21]. Another feature of the focus group is its spontaneity. It provides a more naturalistic setting to stimulate the progress of the ideas exchanged.

There are usually six steps in conducting a focus group discussion [3]. Before the first step, researchers need to decide on the group’s size and the members’ diversity. As the participants arrive, the facilitator can firstly welcome them for coming and then outline the scope of this discussion. It is also necessary to indicate the ground rules such as participants can step in to express themselves at any time, and that opinions are not right or wrong. Each participant’s individual introduction serves the purpose of building an initial rapport among them and provides background information for analysis. It is best for the opening topic to be neutral and general to prompt the discussion. During the main body of the discussion, the facilitator needs to maintain a balance between free-flowing discussion and covering all research relevant issues. To avoid an abrupt finish, the facilitator can signal in advance about the discussion beginning to close [3]. It is helpful to inform the participants about the data management method after the discussion. 

#### 3.3.3. Observation

The observational method is particularly suitable in following several situations where data is not entirely accessible through other methods: (a) discrepancies exist between what people do and what they say [1]; (b) naturally occurring and unconscious behaviours and (c) complex interactions involving an environment or physical context.

There are usually six steps in conducting an observation. Firstly, there is a range of issues to consider when selecting the research fields and gaining access to them [1,4]: (a) salient features relevant to the research topic, (b) observer and participants’ familiarization with sites, (c) the participants’ basic characters, (d) the time and frequency of observation, and (e) different priories according to different gatekeepers. The next step is to identify the participants. Before the observation period commences, it is vital to complete the background information such as the time, venue, observer, etc. During the observation, data recording can be in the form of fieldnotes, diagrams or visual records. Since it is difficult to record every detail because observation is ongoing, some researchers will choose to jot down their fieldnotes first. It is essential to establish an explicit indexing and logging system to manage these raw materials [3]. Before closing the observation, it is worthwhile recording any appeared sign for further activities. Due to the flexibility of the observation study design, the exact steps of an observation will vary depending on the research questions. Post-observation notes can be generated from the observer’s or participants’ perceptions. It is also worth noting that the researcher should not immerse themselves too much in the setting, which is called “going native.” “Going native” can cause a severe consequence in that they can prevent the research objectives being discerned [9].

### 3.4. Data Analysis

Qualitative research usually yields a large amount of data to analyse since the collected raw data only provides a description [4]. Interpreting and explaining data require the researcher to be able to do systematic searching and diligent detection. Data analysis often starts during or immediately after the data collection method to identify new themes for further investigation and sometimes also to refine the research questions [22]. Unlike quantitative research where statistical analyses are often used, qualitative research deals with enormous non-numerical data. That requires the researcher to employ a clear coding and indexing system in addressing the overall research questions. There are also computer-assisted qualitative data analysis (CAQDAS) packages that can help to organize, code and sort large amount of data [23].

**Figure 2:**
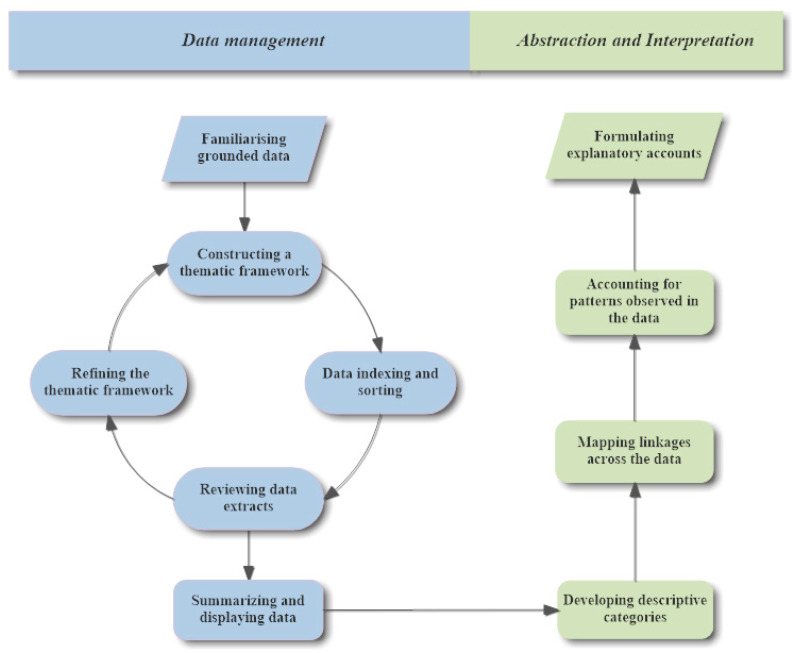
Formal data analysis process of qualitative research (Adapted from Ritchie et al., 2003 [3]).

Broadly speaking, there are two approaches to qualitative data analysis [24]: the deductive approach (framework analysis) and inductive approach (thematic analysis). Researchers need to choose a suitable data analysis method based on their research questions and the nature of their data. The deductive approach typically has been used in studies in which the researcher already had a predetermined framework to analyse the data. In comparison, the inductive approach derives the thematic framework from actual collected data rather than the predetermined framework. Thematic analysis is the most commonly used method in dental qualitative research [25]. The formal analysis process (Figure 2) consists of two main stages and 10 key steps [3]. 

In the data management stage, familiarization requires researchers to have an overview of the relevant content and topics within the data. To achieve this, researchers can conduct open coding on the transcripts by labelling detected phrases and making notes in the margins of transcripts [22]. An initial thematic framework can be constructed by listing, reducing and grouping previously detected topics into a set of themes and subthemes. Then the initial thematic framework can be used to index and sort all of the data obtained. Reviewing the data extracts facilitates refining the initial thematic framework. Generating a final thematic framework is a cyclical working process. Researchers are recommended to summarise and display their data extracts in a set of matrices. After data management, researchers need to tease out what will be the final findings by developing categories of extracted data, mapping the linkage within defined categories and explaining the particular way of linking [3].

### 3.5. Reporting Qualitative Research

Composing a qualitative research paper is similar to quantitative research: both consist of a title, an abstract, an introduction, objectives, methods, findings and discussion. The difference is that a qualitative research paper will be less regimented than quantitative research. Unlike quantitative research that provides hypotheses before testing, the hypotheses in qualitative research are generated through inductive reasoning based on the data collected [16]. The participants’ profiles will be detailed in the method section, along with the overview of the interview/discussion guide. The data analysis method is also stated with the underlying theoretical perspectives [23].

Decisions about how to report and discuss qualitative research findings should be guided by methodological positions underpinning the research topic [3]. The reporting format can be comprehensive, a summary, developmental or selective. No matter which format is chosen to report the findings, key challenges include how to structure the findings logically, map the coverage and diversity, appropriately display the illustrative material and combine qualitative findings with quantitative results. There are broadly two ways for writing the results and discussion parts of a qualitative research paper [26], namely (i) reporting key findings under each theme, followed by a separate discussion, and (ii) combining key findings and discussion into one section. When displaying the key findings, different levels of coding and how the information extracted led to the accounts should be described [23]. Depending on the types of data collected, different uses of illustrative material can be chosen, such as quotations, summaries, sections of researchers’ observational notes and photos.

### 3.6. Ethical Consideration in Qualitative Research

The interactive and flexible nature of qualitative research will inevitably increase the occurrence of ethical dilemmas. Consideration of possible ethical issues and the solutions to address them should start from early stages of study design [27]. For example, the data collection should be based on informed consent; the participants should be given the assurance of confidentiality and anonymity; the undue intrusion should be avoided with proportionate probing and exploration, etc. Researchers need to think from the perspective of their participants and establish a good rapport with them to conduct a high-quality qualitative research [3].

## 4. Functions of Qualitative Research in Dentistry

Compared with quantitative research, qualitative research can probe underlying values, beliefs and attitudes. In recent years, qualitative research has become popular in dentistry because it reveals insights that cannot be captured by quantitative research. The insights may be about the relationships between professional groups (i.e., dentists and dental teams) or between dental personnel and care receivers. To explore these social interactions, long-term observations and in-depth interviews can enhance the findings’ quality [28]. Qualitative research can offer insights into one’s lived experience as well (i.e., treatment experience). To achieve a higher level of understanding, researchers need to deeply focus on the meaning that participants attached to their experiences [27]. Besides, qualitative research is appropriate when investigating attitudes towards new policy, management or practices. The systematic watching and listening of participants in their natural setting can provide researchers useful information or different perspectives. There are four potential functions of qualitative research in dentistry: contextual, explanatory, evaluative and generative. These four functions are neither exhaustive nor mutually exclusive in that they can be used during different stages of the dental research process with different purposes. Table 4 displays some examples of dental research that contribute to each of these functions.

### 4.1. Contextual

Contextual qualitative research is valuable in providing information about the form and nature of existing phenomena [3]. This kind of qualitative research allows the study population to describe how they perceive and understand the research topic in their own terms. Therefore, differing perspectives with specific details of social phenomena can be unpacked and explored [41]. Contextual qualitative research can provide descriptive and exploratory evidence in different ways, including defining the dimensions within a phenomenon, describing the features of the phenomenon, exploring the meaning that participants attached to the phenomenon and mapping the typologies. For example, the oral health beliefs among the Bulang ethnic minority group in China or how residents in rural Quebec perceive their oral health may not be easily revealed in epidemiological surveys, but can be explored in qualitative studies [29,31].

### 4.2. Explanatory

Given the facility to probe underlying values and beliefs, explanatory qualitative research can seek out the factors or influences that drive the occurrence of a specific phenomenon or associations between social phenomena [3]. It is also effective in identifying the motivations for people’s actions, which may indicate some explanatory or causal links between people’s thinking and decisions. Moreover, explanatory qualitative research can explore the context in which the phenomena occur, for example, it could investigate children’s quality of life in the context of living with cleft lip and palate [36]. Explanatory research can provide evidence in identifying the critical influences and in generating a higher level of understanding than contextual qualitative research.

### 4.3. Evaluative

Evaluative qualitative research is more concerned with issues related to policy making or organizational investigation. In dentistry, this kind of qualitative study is particularly adept at evaluating the performance of healthcare programs, services, products or interventions. Qualitative research can provide evidence on both the process (factors that shape a programme or service) and the outcomes (different types of effects or consequences). From this definition, it is possible to classify evaluation modes into formative evaluations and summative evaluation [42]. Formative evaluations seek the information to improve the programme, including the programme’s details, the dynamics of how things operate within the programme, theories underpinning the programme, the target population’s requirements and factors contributing to the programme’s successful delivery. In comparison, summative evaluations emphasize the different impacts of the programme including the effects of taking part in the programme and the influence of contexts in which interventions are provided on the programme’s effectiveness [3].

### 4.4. Generative

Generative qualitative research is useful for developing and generating new theories, concepts and hypotheses. It can also be effective in generating new strategies or solutions to persistent problems [1]. Knowledge generation is a collaborative process involving all participants with their own experiences and understanding. Thus, the key features of qualitative research allow the original and creative thoughts to be raised from the natural settings. The theories or strategies developed through this approach can have more benefits for those populations involved in the research, and the outcome will also have a more comprehensive application [43].

## 5. Conclusions

Qualitative research can provide depth and detail, create openness, simulate people’s individual experiences and attempt to avoid pre-judgements. Dental researchers need to ensure that qualitative studies are constructive and methodologically rigorous to maintain the clarity of data analysis. Effective qualitative research allows a greater spontaneity of views the participants raise and possesses the ability to continue probing for more nuanced information. Qualitative research possesses its own potential functions in serving different stages of the dental research process.

## Figures and Tables

**Table 1:** Domains of quantitative research and qualitative research (Adapted from Ritchie et al., 2003; Masood et al., 2010; Bower et al., 2007 [3,4,5]).

Domain	Quantitative Research	Qualitative Research
Knowledge Acquisition	Deductive reasoning to confirm hypotheses	Inductive reasoning to explore phenomena
	Objective	Subjective
Analytical objectives	To quantify variation	To describe variation
	To predict causal relationships	To describe and explain relationships
	To describe characteristics of a population	To describe individual experiences/group norms
	Fixed	Allowing for refinement
Study design	Stable from beginning to end	Flexible and iterative
	Little interaction between participant and researcher	Frequent interaction between participant and researcher
	Contextual factors are often eliminated in controlled studies	Context is essential in shaping meanings and explanations
Sampling	Probability sampling	Purposive sampling
	Representativeness of population	Diversity of population
	Sample size determined by power calculation	Sample size determined by theoretical saturation
Methodology	Well-determined methods	Flexible methods
	Predetermined and rigid style of eliciting and categorizing responses	Flexible and iterative style of exploring emerging response
	Largely depends on measurements device or instrument	Largely depends on the researcher’s skill and rigor
Data format	Numerical—assigning numerical values to responses	Textual—audiotapes, videotapes and field notes
	More breadth on numerous cases	More in-depth on a few cases
Data Analysis	Statistical tests	No statistical tests
	Analysis of variables	Analysis of themes
	Value-free analysis	Shaped by researcher’s value
Output	Descriptive statistics	Detailed description, explanations, classifications and typologies
Generalizability	Statistical generalization	Representational, inferential or theoretical generalization

**Table 2:** Data collection by in-depth interview, focus group and observation (Adapted from Ritchie et al., 2003; Masood et al., 2010; Bower et al., 2007 [3,4,5]).

Data Collection Method	In-Depth Interview	Focus Group	Observation
Data generation	One-on-one interview	Group discussion	Systematic watching
When to use	Exploring issues in depth and detail	Generating data shaped by group interaction	Exploring what actually happens
	Exploring complex processes and issues	Displaying a social context	Observing naturally occurring and subconscious behaviours
	Exploring private issues or subjects	When creative thinking is required	Exploring public behaviours with environment involvement
Study population	For participants less willing or able to travel	For participants willing and able to travel	For participants who will be engaged in a public setting
	Where the participants are geographically dispersed	Where the participants are geographically clustered	Where the participants interact in a particular setting
	Where the participants are highly diverse	Where the participants have some common ground	
Recording method	Fieldnotes	Fieldnotes	Fieldnotes
	Audiotapes	Audiotapes	Diagrams
	Audio-video tapes	Audio-video tapes	Visual records

**Table 3:** Steps in conducting in-depth interview, focus group and observation (Adapted from Ritchie et al., 2003 [3]).

Step	In-Depth Interview	Focus Group	Observation
1	Arrival and introduction	Setting scenes and grounded rules	Site arrangement
2	Research introduction	Individual introduction	Identifying the participants
3	Beginning the interview	Opening topic discussion	Collecting background information
4	During the interview	During the discussion	During the observation
5	Ending the interview	Ending the discussion	Closing the observation
6	Post interview	Post discussion	Post observation notes

**Table 4:** Examples of qualitative studies with contextual, explanatory, evaluative and generative functions.

Examples of Studies	Domain	Objectives of the Studies	Data Collection	Data Analysis
**Contextual Functio**				
Zhang et al., 2018 [29]	Defining dimensions	To explore traditional oral health beliefs among the Bulang ethnic minority group in China	Focus groups	Thematic analysis
Shahnavaz et al., 2015 [30]	Describing features	To explore how children with dental anxiety and their parents experience cognitive behavioral therapy in dentistry	In-depth interview	Thematic analysis
Emami et al., 2014 [31]	Exploring meaning	To explore how residents in rural Quebec perceive their oral health and their access to dental care	Semi-structured interviews	Thematic analysis
Stein et al., 2019 [32]	Mapping typologies	Explore caregivers’ and dentists’ approaches to improve oral care for children with autism	Focus group	Thematic analysis
**Explanatory Function**				
Moore et al., 2004 [33]	Factors underline a particular perception	To seek the contributing role of embarrassment to phobic dental anxiety	In-depth interview	Framework analysis
Liu et al., 2019 [34]	Motivations lead to decisions	To identify barriers to and motivators for dental care-seeking behaviours of pregnant women	Semi-structured interviews	Thematic analysis
Muirhead et al.,2013 [35]	Origins of experiences	To understand why low-income parents may underutilize free dental services.	In-depth interview	Thematic analysis
Zeraatkar et al., 2019 [36]	Contexts in which phenomena occur	To investigate children’s quality of life in the context of living with cleft lip and palate	In-depth interview	Thematic analysis
**Evaluative Function**				
Ajwani et al., 2019 [37]	Formative evaluation	To undertake a process evaluation and explore the perceptions of dental professionals involved in the Midwifery Initiated Oral Health Dental Service	Focus groups	Framework analysis
Mariño et al., 2005 [38]	Summative evaluation	To evaluate an oral health promotion program for older migrant adults	Focus groups	Thematic analysis
**Generative Function**				
Bedos et al., 2003 [39]	Developing concepts or hypotheses	To investigate the dental care pathway of welfare recipients in Quebec	In-depth interview	Thematic analysis
Luo et al., 2018 [40]	Generating strategies or solutions	To develop an instrument to assess dental satisfaction	Focus groups	Thematic analysis

## Data Availability

Not applicable.
